# Hemothorax an Unusual Complication during Liver RFA

**DOI:** 10.1155/2011/329491

**Published:** 2011-12-20

**Authors:** Paraskevi Tsagouli, Mary Pomoni, Savvas Tanteles, Anastasia Pomoni, Loukas Thanos

**Affiliations:** Evgenidion University Hospital of Athens, Research Center of Radiology and Imaging, 11538 Athens, Greece

## Abstract

We present a case of a 70-year-old patient with hepatocellular carcinoma treated with RFA. The lesion was located in segment II under the ribs. During RFA pleural effusion is presented. After the procedure a dual phase CT revealed haemothorax and extravasation of the contrast medium from the intercostal vessels.

## 1. Introduction

Percutaneous radiofrequency ablation is used for treatment of hepatocellular carcinoma and it can be performed with a variety of imaging modalities ultrasound, computed tomography, magnetic resonance imaging, and fluoroscopy. Severe complications can happen like intraperitoneal bleeding, pneumothorax, hemothorax, liver abscess, and bile duct injury. In our department RFA is performed under CT guidance and complications can be detected with accuracy. In our case we present a case of a female patient with HCC who complicated with pleural effusion detected with CT. Intravenous contrast medium administration revealed a jet-like extravasation from the intercostal vessels which is evolved to hemothorax.

## 2. Case Presentation

**Figure 1:**
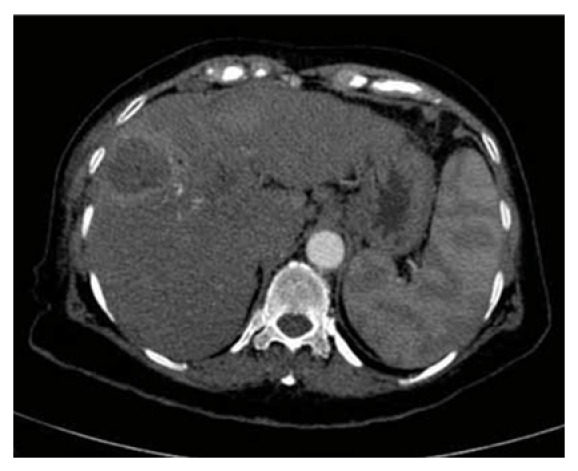
A 70-year-old woman with two lesions of hepatocellular carcinoma. Contrast-enhanced CT scan shows two hepatocellular masses in segment V (treated before with RFA) and II, under the ribs.

A 70-year-old woman with two lesions of hepatocellular carcinoma was treated in our department. The first large lesion, located in segment V, was ablated several times the previous years. A new lesion appeared in segment II, located under the ribs (Figure 1), which was decided to be treated with RFA.

Prior to therapy the patient had underwent laboratory examinations: hematocrit, white blood cell count, blood coagulation tests, values for hepatic, function and a-fetoprotein levels. Platelet (PLT) count value was 60,000/mL and international normalized ratio (INR) was 1,2. Forty-five minutes before the procedure the patient received analgesic and antidepressant treatment consisting of one pill of 3 mg bromazepam per os and 75 mg d-propoxyphene hydrochloride intramuscularly. CT-guided RFA started by placing the patient in the supine position. The shortest, most vertical, and safest path should be chosen. The front path just over the lesion was chosen. The skin at the needle entry site was prepared with povidone iodine 10% solution. A 22 G needle for syringe use was inserted into the skin, and three contiguous CT images were obtained to ensure that the chosen point was the appropriate one. Local anaesthetic (2% lidocaine hydrochloride) was then instilled through this needle for skin and subcutaneous tissue anaesthetization. The needle was removed and an incision with a surgical blade was made to facilitate electrode cannula insertion. Ablation was carried out with a MIRAS triple spiral 15 G electrode.

**Figure 2:**
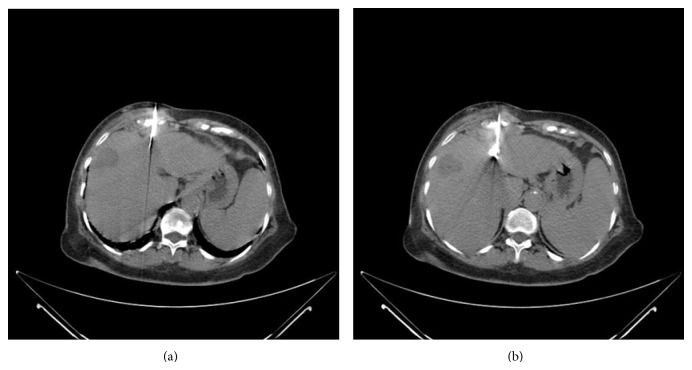
The front path just over the lesion was chosen. The tip of the electrode was positioned in the correct position and it was deployed slowly.

The tip of the electrode was positioned in the correct position and it was deployed slowly (Figure 2). A pulsed RF energy was applied for 10 minutes.

**Figure 3:**
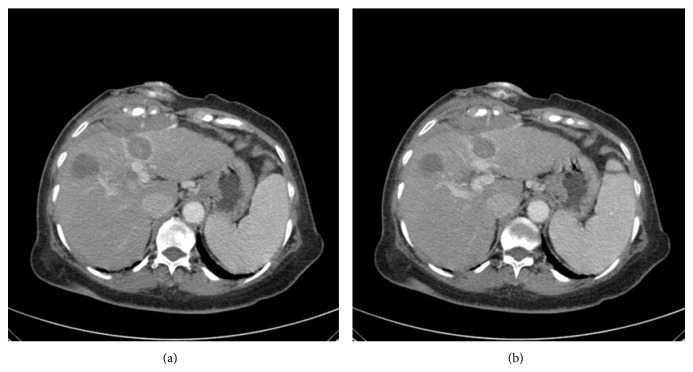
A dual-phase dynamic contrast-enhanced CT was performed just after the RFA procedure which revealed a jet-like extravasation from the intercostal vessels.

**Figure 4:**
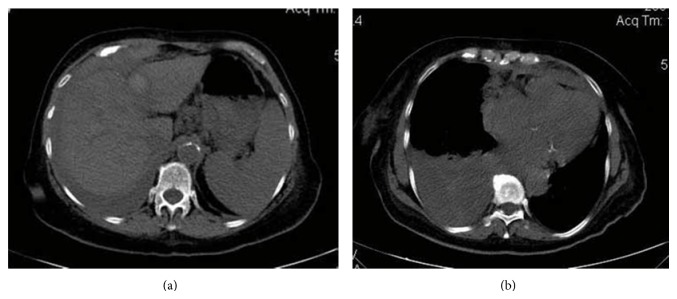
A chest CT was performed immediately which revealed a right pleural effusion with a distinct fluid level.

A few minutes before RFA, CT scan showed pleural effusion, and right after the ablation of the lesion was completed, low pulsed RF energy was applied for the ablation of the track to avoid tumor seeding and ablate the source of the bleeding. A dual-phase dynamic contrast-enhanced CT was performed which revealed a jet-like extravasation from the intercostal vessels (Figure 3). Additionally the tumor was imaged as low-density area with ring enhancement. The patient was haemodynamically stable and a chest CT was performed immediately which revealed a right pleural effusion with a distinct fluid level (Figure 4). Drainage was performed under CT guidance in order to identify the fluid and monitor the blood loss. The patient continued to be hemodynamically stable and was hospitalised for a few days.

## 3. Discussion

According to a study Hyunchul Rhim et al. [1] reported that hemothorax is one of the major complications that can occur after radiofrequency thermal ablation of hepatic tumors (<0.13%). They underline that a safe window of needle positioning by a skillful operator is the only way to avoid such complications and interventional treatment for pneumothorax or hemothorax should be considered on a case-by-case basis. They present a case of a 47-year-old woman with hepatocellular carcinoma who underwent RF ablation of the tumor in segment VII of the liver. Chest CT scan was obtained after RF ablation which revealed fluid collection of relatively high attenuation in the right hemithorax, a finding indicative of hemothorax [1]. The hemothorax was self-limited, as in our case.

Goto et al. [2] in an analysis of the risk factors and management of hemorrhagic complications of RFA treatment, presented 14 cases of hemothorax in total 4133 treatments (0.3%). Three patients required both blood transfusion and thoracic drainage, whereas 2 other patients received blood transfusions only. All of the other patients recovered with conservative treatments only. Common symptoms were chest pain and dyspnea. However, about half of the cases of hemothorax were asymptomatic and detected during a routine CT taken to evaluate the efficacy of RFA. They analyzed the risk factors for each hemorrhagic complication. The location of the tumor nodule was a significant risk factor for hemothorax (segment 7, odds ratio OR = 2.31). They emphasise that general measures to prevent complications in RFA include the use of high-quality imaging devices, the assessment of bleeding tendency, availability of platelets or fresh frozen plasma transfusion when indicated, and the careful selection of the needle insertion route and body position during the procedure. In our case we should have chosen a more safe route of needle insertion, to prevent injury of intercostal vessel.

In their opinion, once hemothorax occurs, the respiratory condition may deteriorate rapidly. Acute respiratory distress syndrome may occur and the resulting thrombocytopenia can aggravate the hemorrhage. In addition to stabilizing the circulation by using infusion and transfusion, thoracic drainage is often required to stabilize the respiratory condition and prevent subsequent acute respiratory distress syndrome. If the estimated blood loss was <300 to 400 mL, they performed thoracentesis, whereas if the estimated loss exceeded 500 mL, they performed a tube thoracostomy.

Akahane et al. [3] in a review of the imaging features, risk factors, and management of major and minor complications of percutaneous RF ablation for hepatocellular carcinoma report a prevalence of hemothorax requiring drainage 0.1% per treatment and <0.1% per session. They conclude that pneumothorax and hemothorax are rare but may be encountered if the treated lesion is near the diaphragm and that pneumothorax and hemothorax are usually self-limited.

Livraghi et al. [4] reported between their complications a case of hemothorax that required drainage.

Sugihara et al. [5] reported a case of 61-year-old woman with a hepatocellular carcinoma located in the subphrenic region, treated by radiofrequency ablation (RFA) under artificial pleural effusion. During RFA, ultrasonography showed a swirling high echoic lesion in the artificial pleural effusion.

Using contrast-enhanced ultrasonography (CEUS), a jet-like extravasation of contrast medium was revealed and pooling of microbubbles in the pleural cavity. It was an active bleeding in the pleural cavity, from a branch of the inferior phrenic artery, which was confirmed by angiography. They embolized the branch using a gelatin sponge with a particle size of 1 mm.

Our treatment in this complication was not so aggressive. Positioning a drainage we monitored the blood loss. It was a self-limited hemorrhage and drainage was withdrawn after a couple of days.
